# Supplementary material for: How the Intrinsically Disordered N-Terminus of Cancer/Testis Antigen MAGEA10 Is Responsible for Its Expression, Nuclear Localisation and Aberrant Migration
Source: Biomolecules. 2023 Nov 24;13(12):1704. doi: 10.3390/biom13121704 (PMC10741916; doi:10.3390/biom13121704)
Supplement: Supplementary file 1 [file biomolecules-13-01704-s001.zip › biomolecules-2723645-original images.pdf]

Original immunoblot images

Figure 1

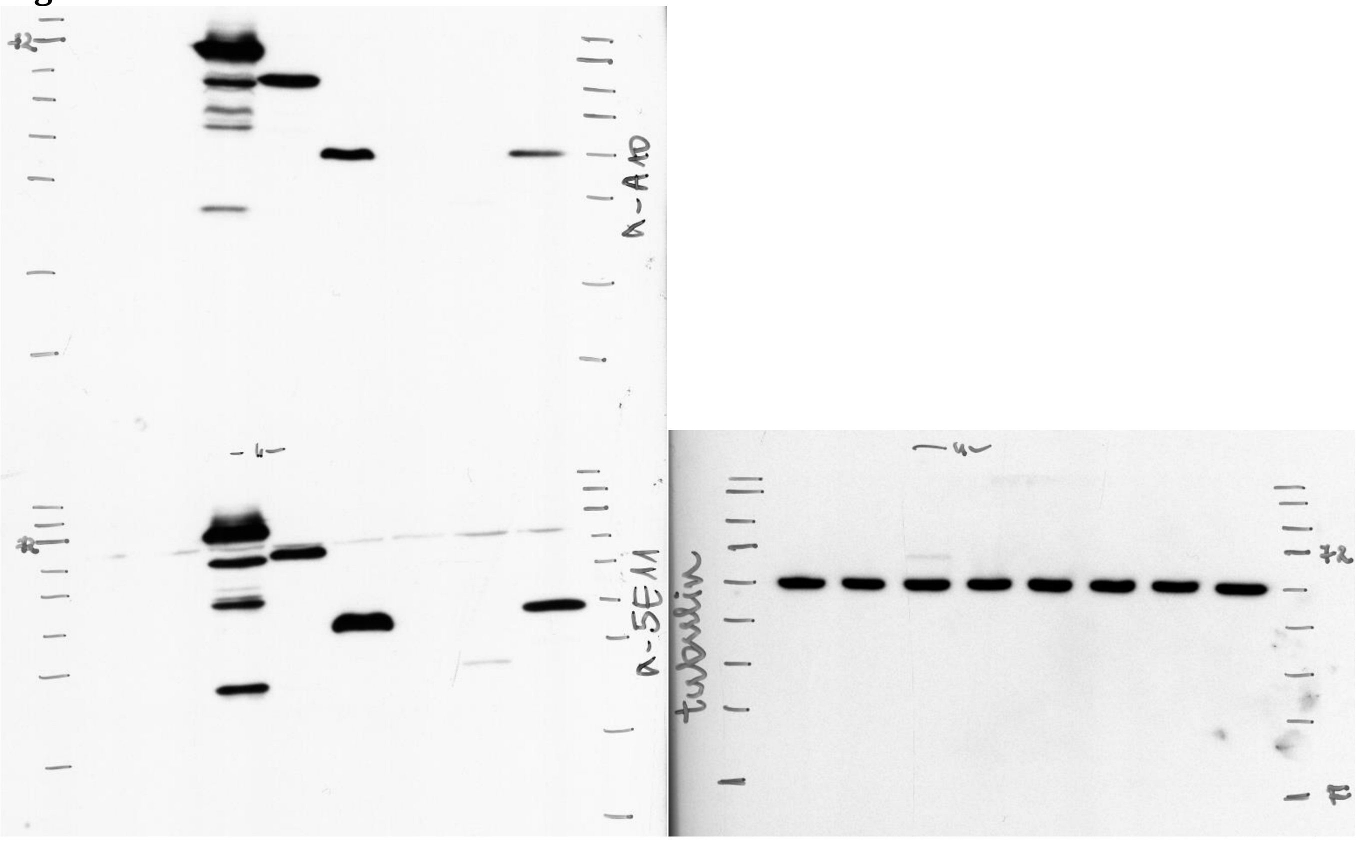

Figure 1C. The upper blot was stained with the anti-MAGEA10, the lower left with the anti-E2Tag 5E11, and the lower right with the anti-tubulin antibody. The first two lanes both depict mock transfections and thus only one is depicted in the paper.

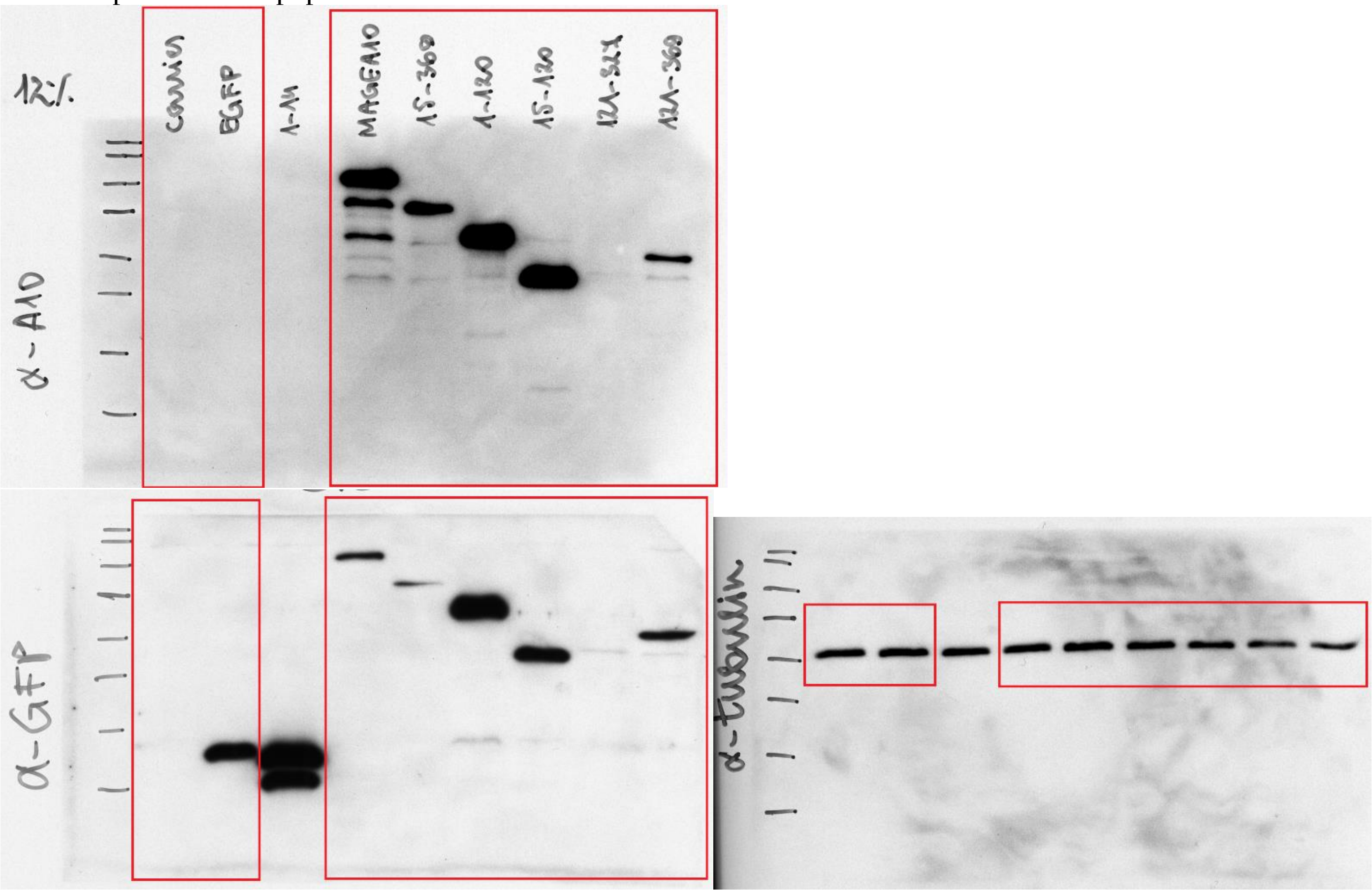

Figure 1D. The upper blot was stained with the anti-MAGEA10, the lower left with the anti-EGFP, and the lower right with the anti-tubulin antibody. The third lane depicting mutant 1–14 containing only the first 14 amino acids of MAGEA10 was omitted in this figure but appears in later figures.

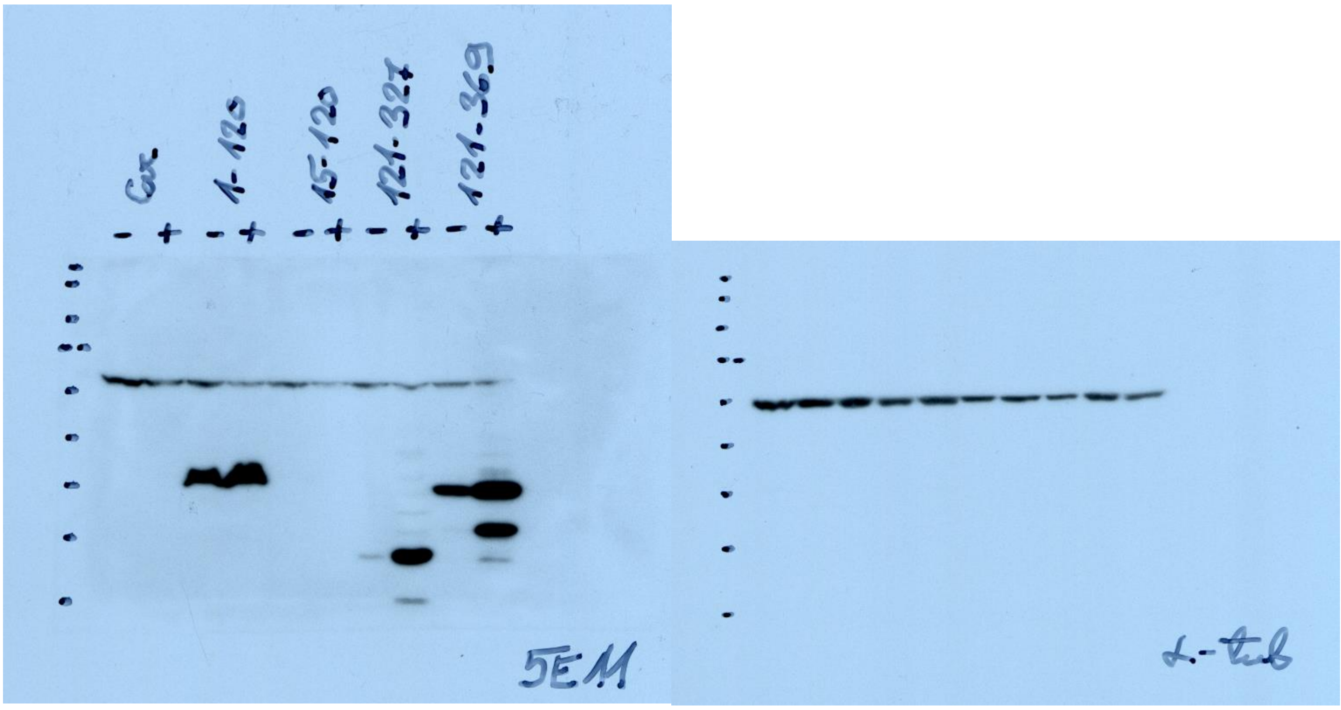

Figure 1E. The blot on the left was stained with the anti-E2Tag 5E11 and the one on the right with the anti-tubulin antibody.

Figure 2

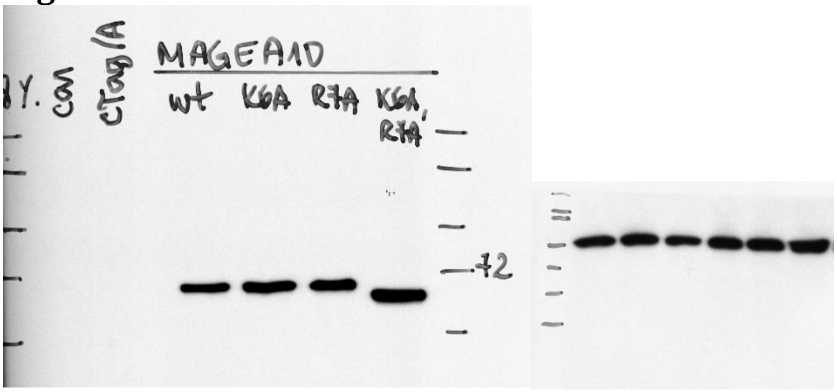

Figure 2B. The blot on the left was stained with the anti-MAGEA10 and the one on the right with the anti-tubulin antibody. The first two lanes both depict mock transfections and thus only one is depicted in the paper.

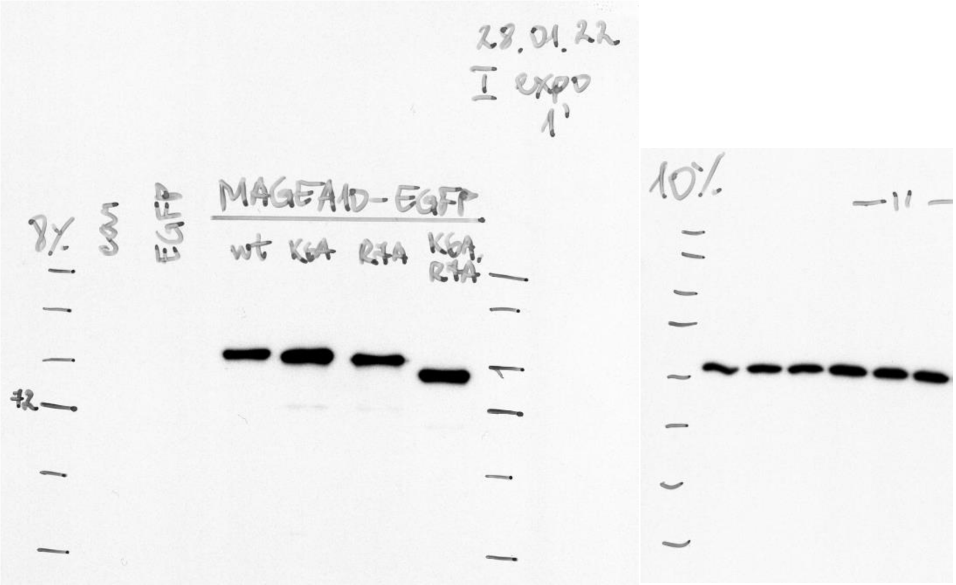

Figure 2C. The blot on the left was stained with the anti-MAGEA10 and the one on the right with the anti-tubulin antibody.

Figure 3

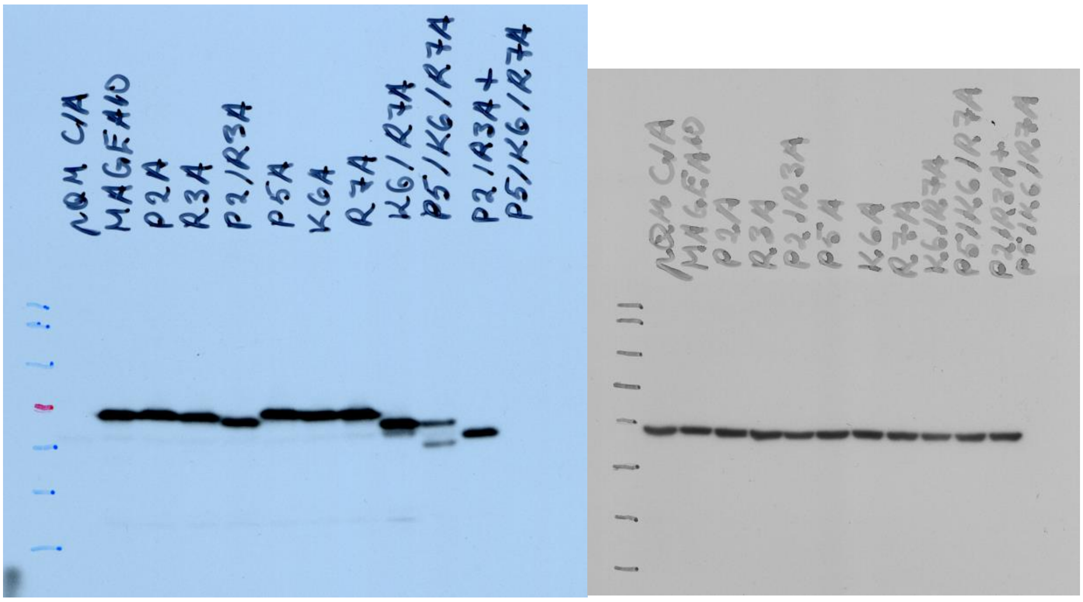

Figure 3B. The blot on the left was stained with the anti-E2Tag 5E11 and the one on the right with the anti-tubulin antibody.

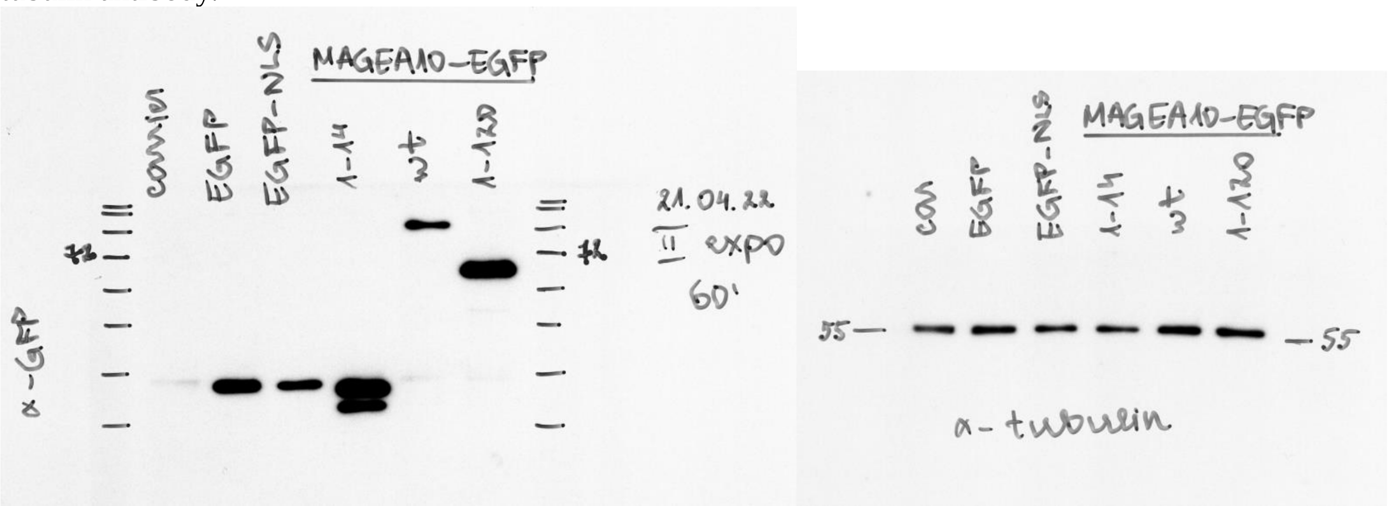

Figure 3D. The blot on the left was stained with the anti-EGFP and the one on the right with the anti-tubulin antibody.

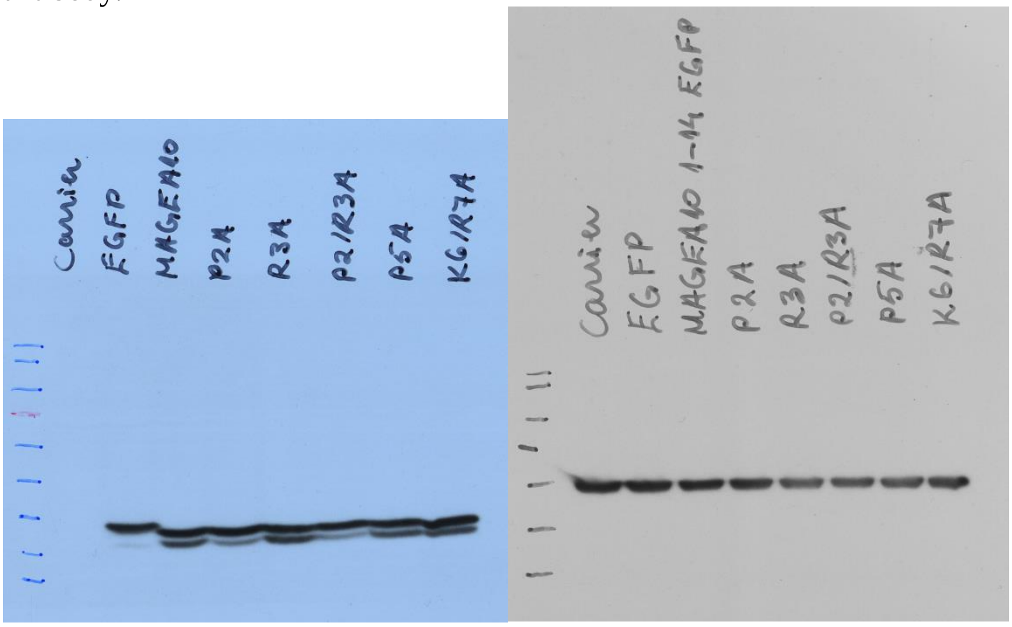

Figure 3F. The blot on the left was stained with the anti-EGFP and the one on the right with the anti-tubulin antibody.

Figure 4

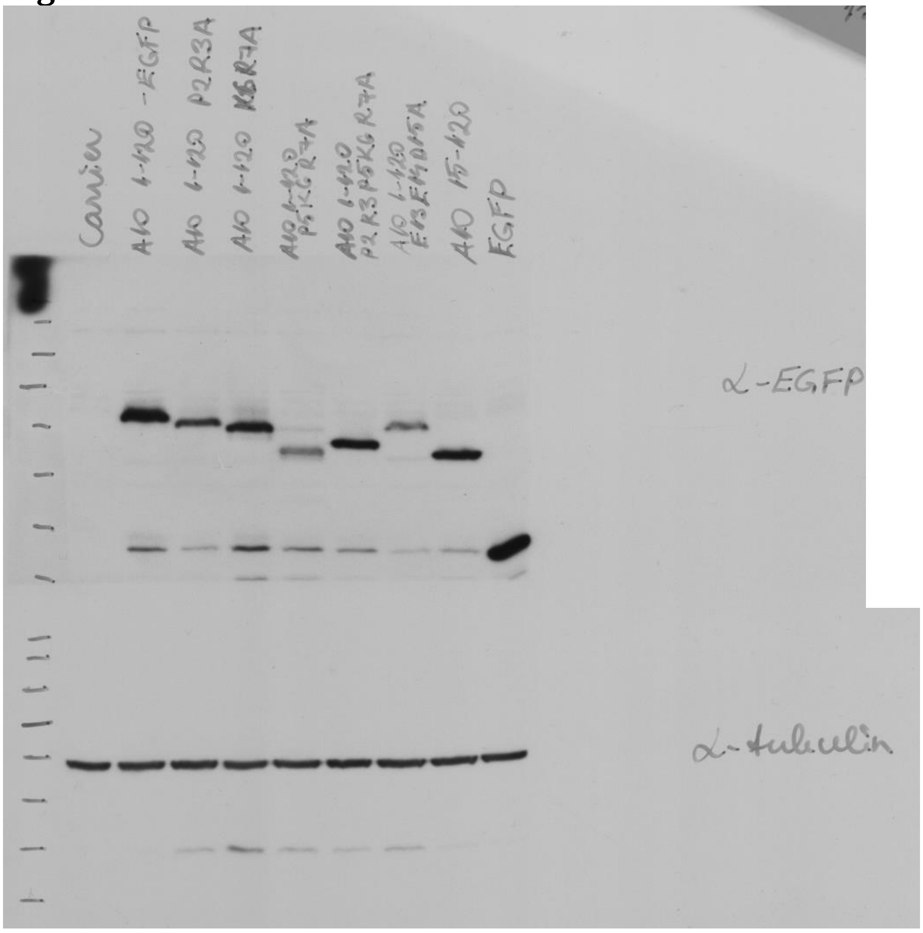

Figure 4B. The upper blot was stained with the anti-EGFP and the lower with the anti-tubulin antibody. The last row depicting pure EGFP was omitted.

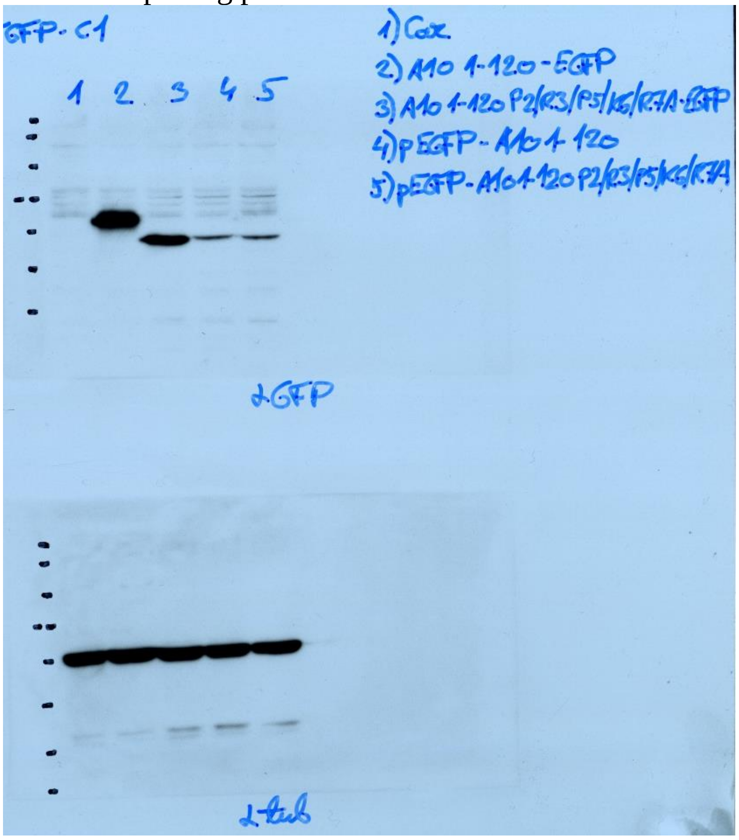

Figure 4D. The upper blot was stained with the anti-EGFP and the lower with the anti-tubulin antibody.

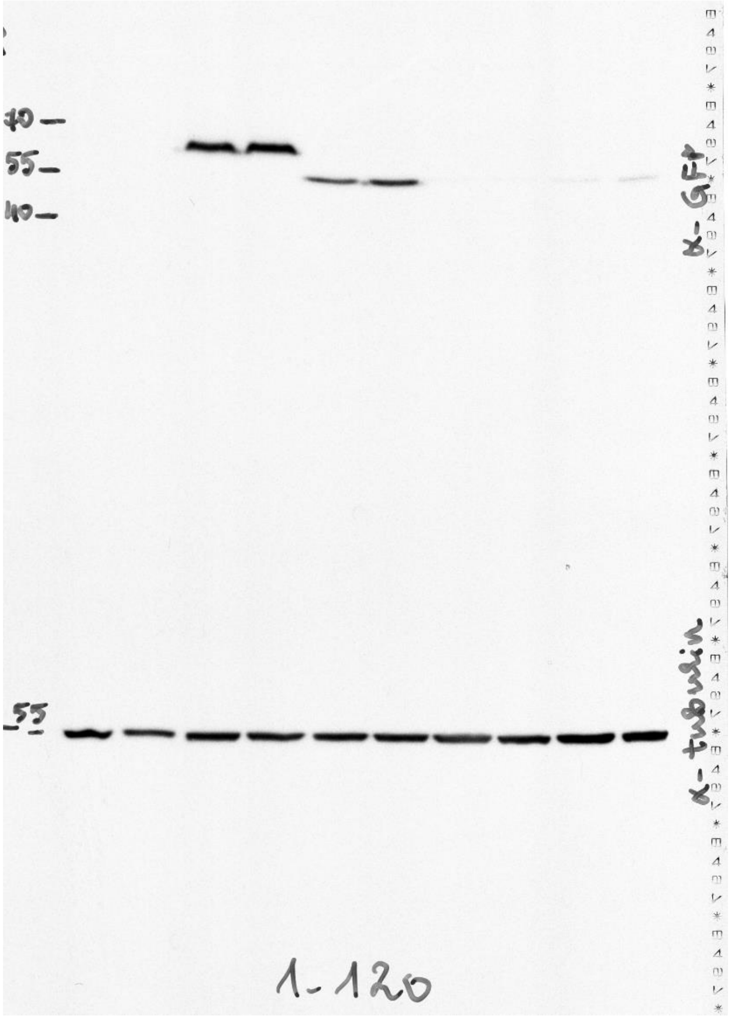

Figure 4F. The upper blot was stained with the anti-EGFP and the lower with the anti-tubulin antibody.

Figure 5

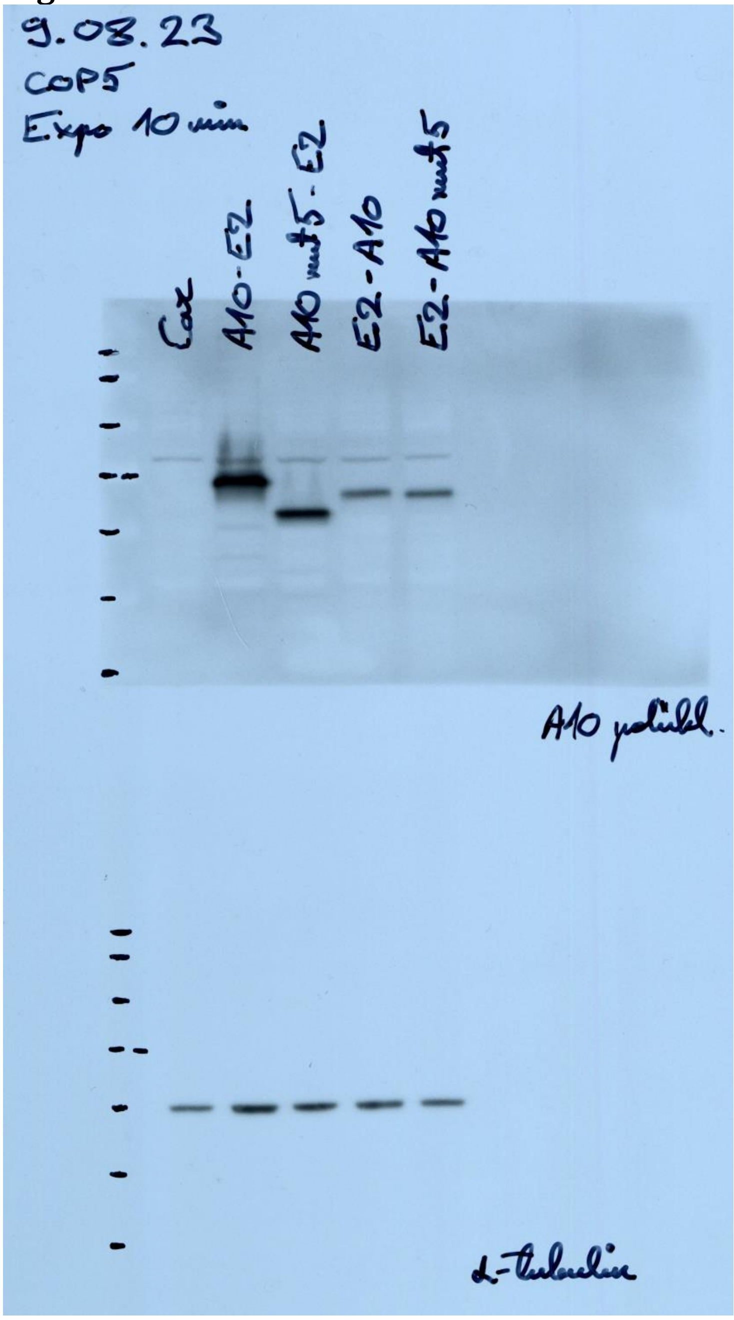

Figure 5B. The upper blot was stained with the anti-MAGEA10 and the lower with the anti-tubulin antibody.

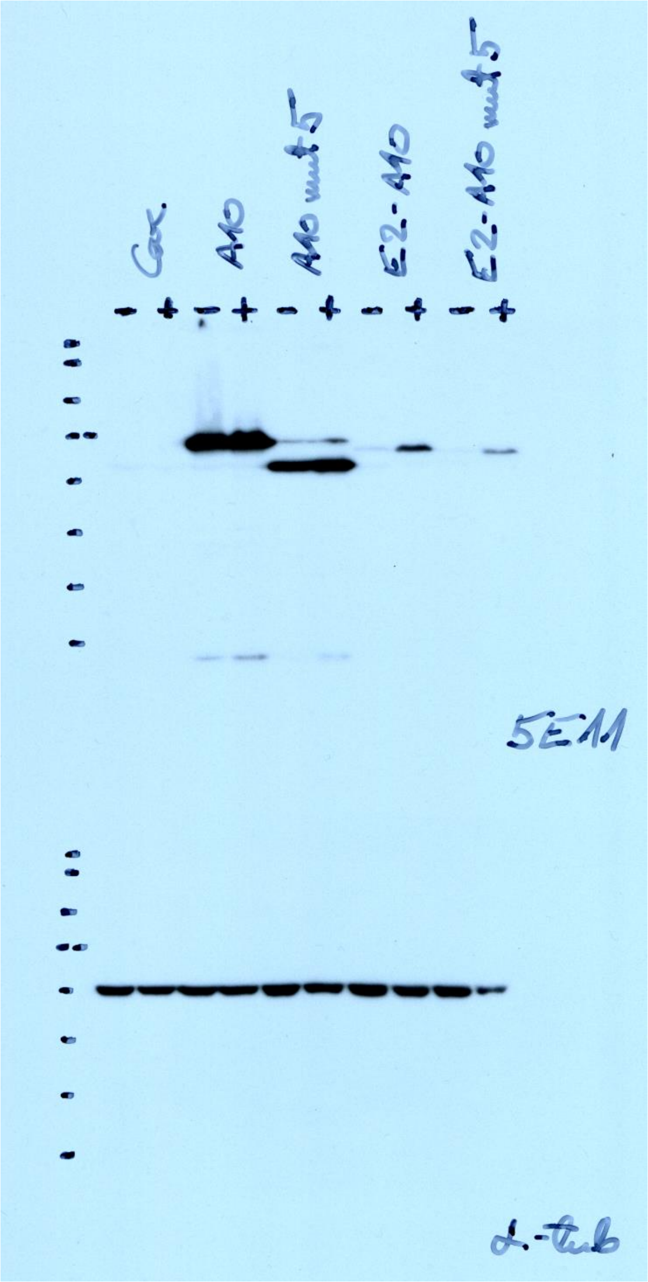

Figure 5C. The upper blot was stained with the anti-E2Tag 5E11 and the lower with the anti-tubulin antibody.

Figure 6

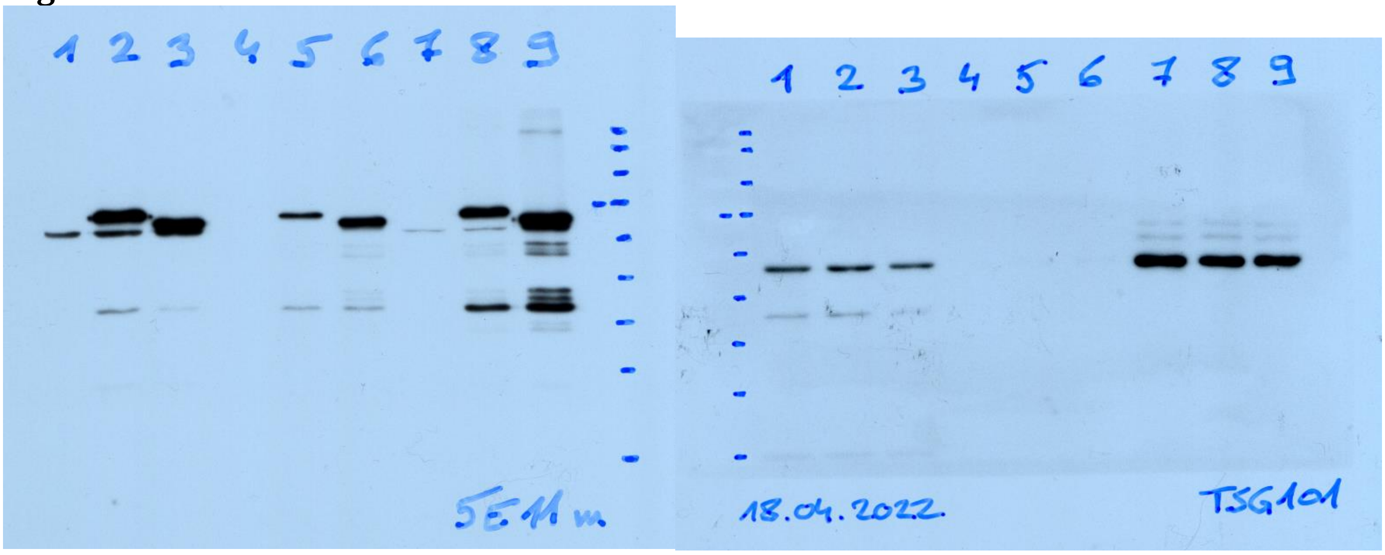

Figure 6C. The upper blot was stained with the anti-E2Tag 5E11 and the lower with the anti-TSG101 antibody.

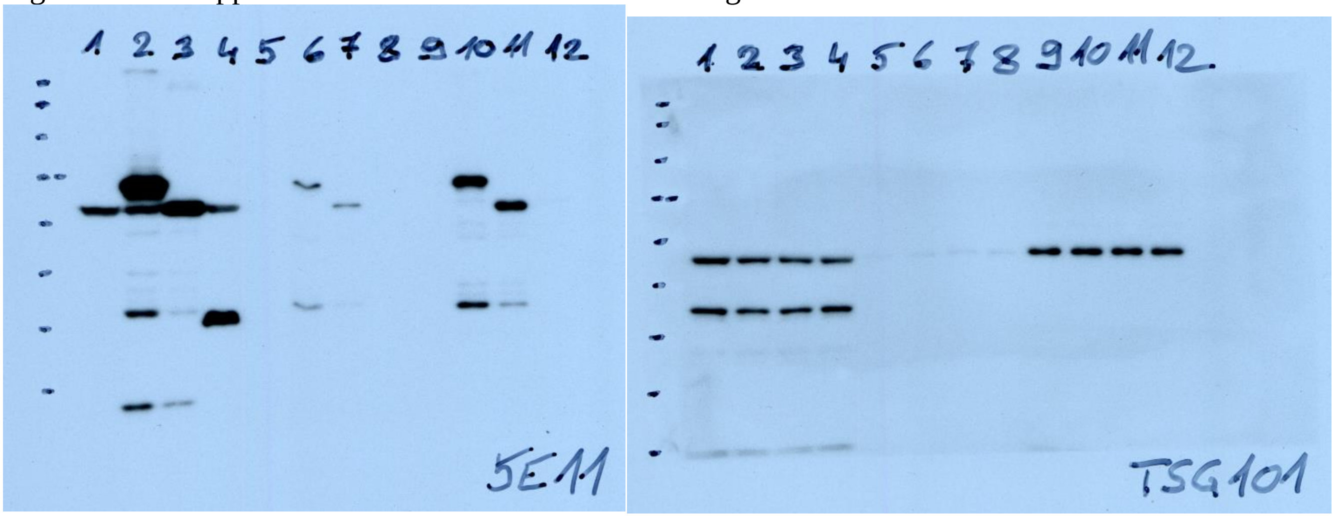

Figure 6D. The blot on the left was stained with the anti-E2Tag 5E11 and the one on the right with the anti-TSG101 antibody.
