# Supplementary material for: How the Intrinsically Disordered N-Terminus of Cancer/Testis Antigen MAGEA10 Is Responsible for Its Expression, Nuclear Localisation and Aberrant Migration
Source: Biomolecules. 2023 Nov 24;13(12):1704. doi: 10.3390/biom13121704 (PMC10741916; doi:10.3390/biom13121704)
Supplement: Supplementary file 1 [file biomolecules-13-01704-s001.zip › Figure S1.pdf]

Figure S1. Quantification of western blot images.

Part 1

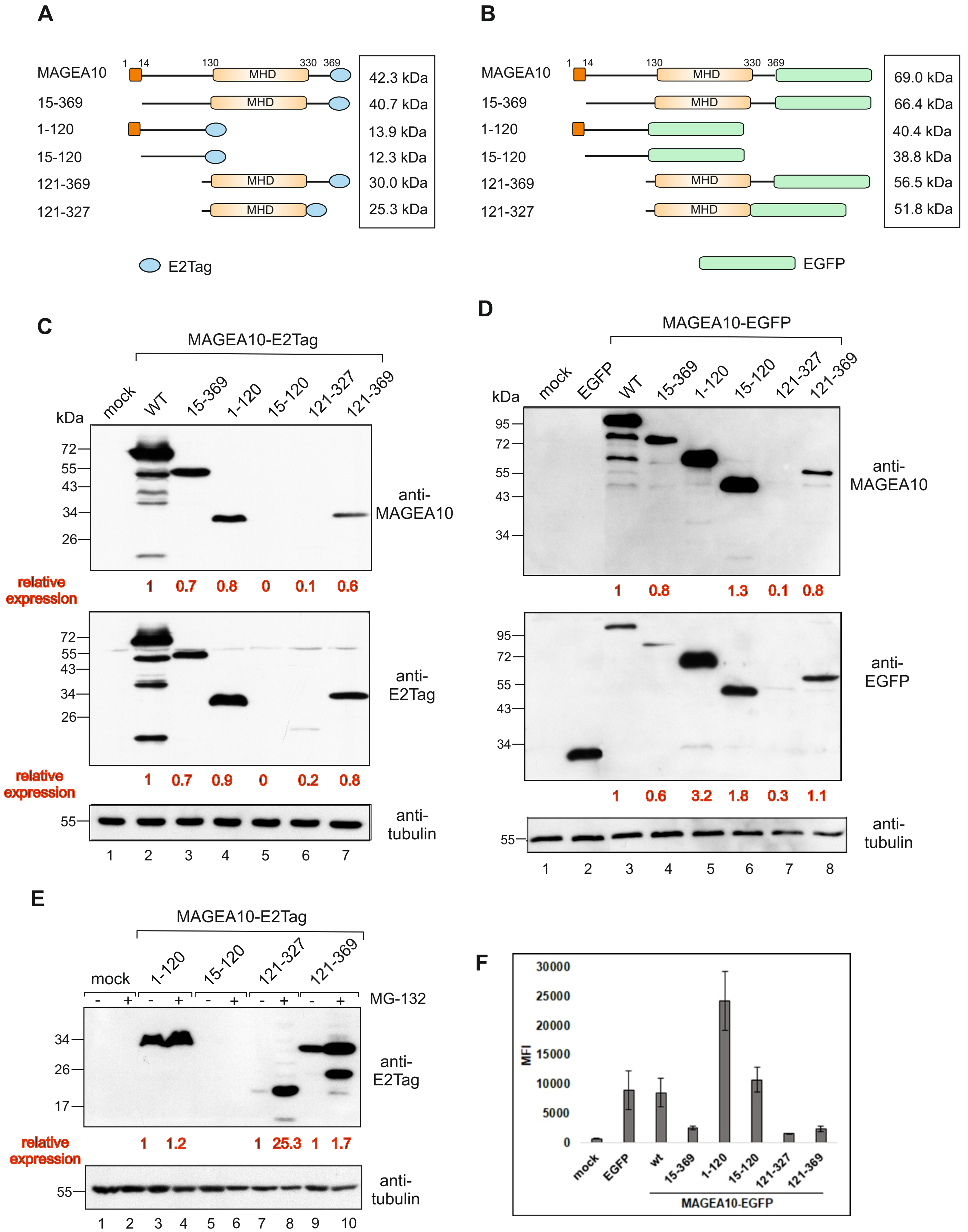

Figure S1. (Part 1) Quantification of western blot images for Figure 1. Quantification was done with Image J software. (C, D) MAGEA10 WT signal was set 1 and the relative expression of deletion mutants is given in arbitraty units. (E) Expression of proteins after MG-132 treatment is given in comparison to without MG-132 treatment.

Part 2

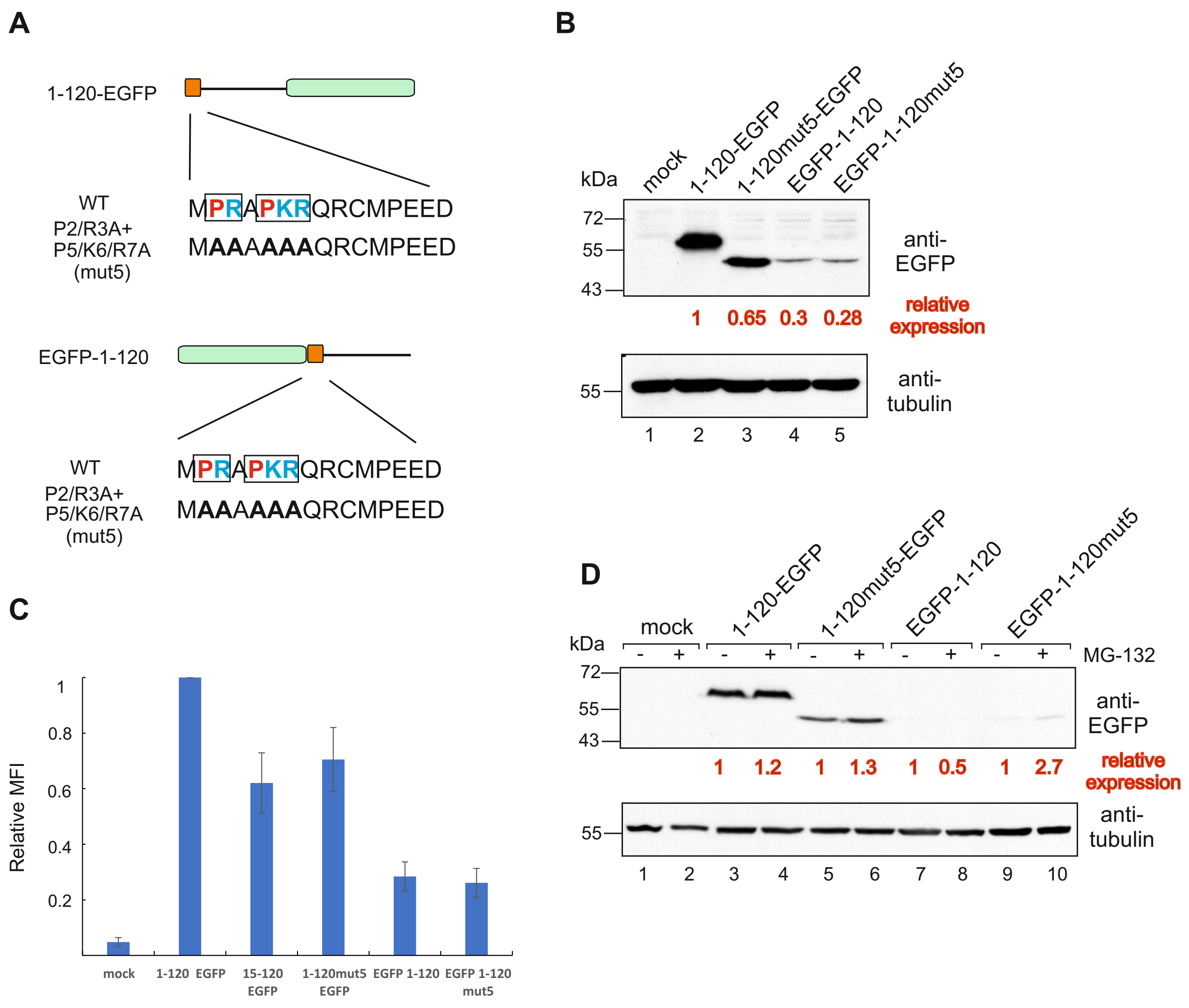

Figure S1. (Part 2) Quantification of western blot images for Figure 4. Quantification was done with Image J software.
(B) MAGEA10 WT signal was set 1 and the relative expression of deletion mutants is given in arbitraty units.
(D) Expression of proteins after MG-132 treatment is given in comparison to without MG-132 treatment.

Part 3

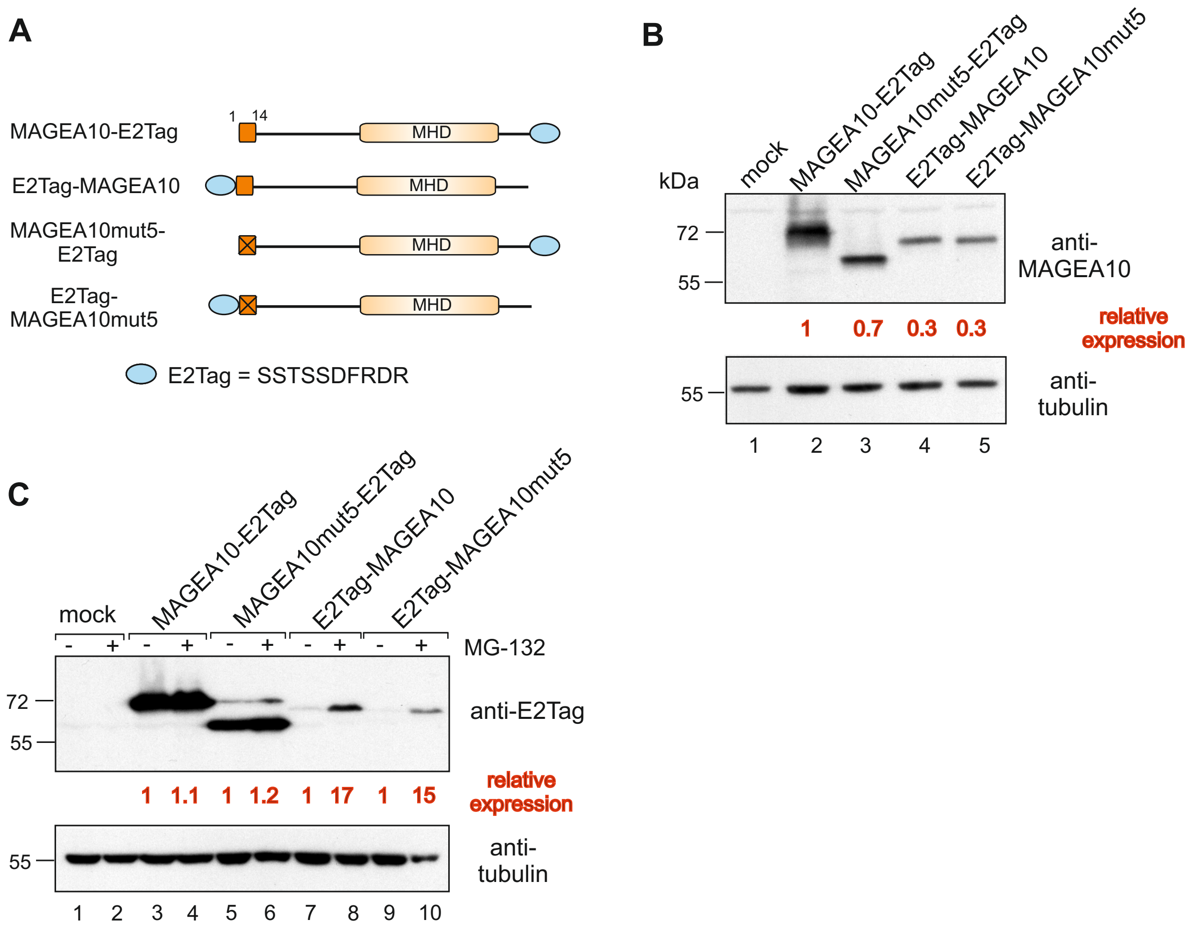

Figure S1. (Part 3) Quantification of western blot images for Figure 5. Quantification was done with Image J software.
(B) MAGEA10 WT signal was set 1 and the relative expression of deletion mutants is given in arbitraty units.
(C) Expression of proteins after MG-132 treatment is given in comparison to without MG-132 treatment.
