# Supplementary material for: How the Intrinsically Disordered N-Terminus of Cancer/Testis Antigen MAGEA10 Is Responsible for Its Expression, Nuclear Localisation and Aberrant Migration
Source: Biomolecules. 2023 Nov 24;13(12):1704. doi: 10.3390/biom13121704 (PMC10741916; doi:10.3390/biom13121704)
Supplement: Supplementary file 1 [file biomolecules-13-01704-s001.zip › Figure S2.pdf]

Supplementary Figure 2

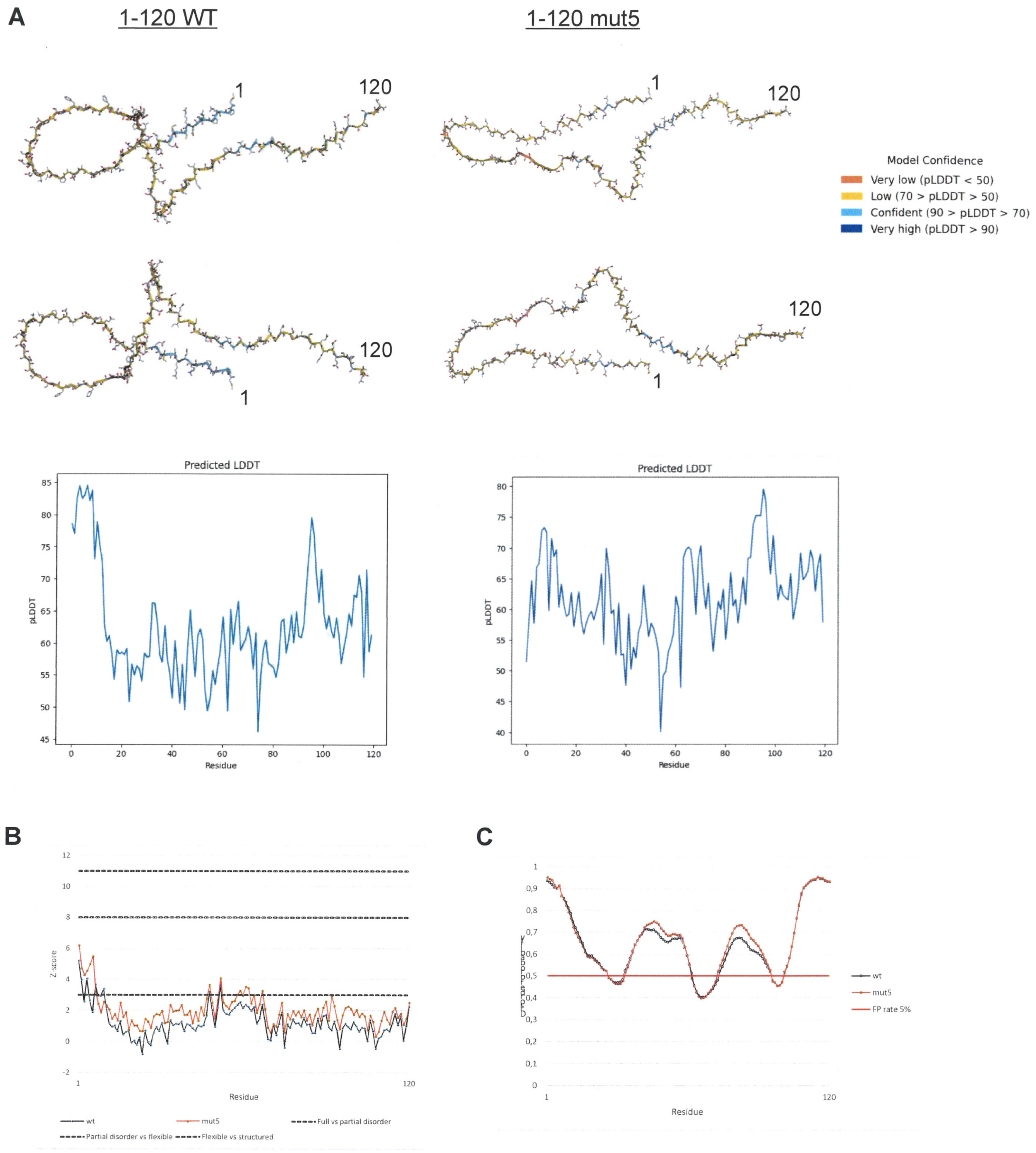

Figure S2. The computational analysis of order/disorder of 1-120 N-terminal residues in WT and mut5 analyzed with three different programs (A) AlphaFold [1], (B) ADOPT [2] and (C) PrDOS [3].

1. Jumper, J., Evans, R., Pritzel, A. et al. Highly accurate protein structure prediction with AlphaFold. Nature **2021**, 596, 583–589, doi: 10.1038/s41586-021-03819-2.
2. Redl et al. ADOPT: intrinsic protein disorder prediction through deep bidirectional transformers. NAR Genom Bioiform **2023**, 5, lqad041, doi: 10.1093/nargab/lqad041.
3. Ishida, T and Kinoshita, K, PrDOS: prediction of disordered protein regions from amino acid sequence., Nucleic Acids Res, **2007**, 35, Web Server issue, W460-464. doi: 10.1093/nar/gkm363.
